# Characteristics of Fungal Communities in Red Mud/Phosphogypsum-Based Artificial Soils

**DOI:** 10.3390/biology14030285

**Published:** 2025-03-11

**Authors:** Yong Liu, Zhi Yang, Lishuai Zhang, Fang Deng, Zhiqiang Zhao, Binbin Xue, Jingfu Wang

**Affiliations:** 1 College of Biological and Environmental Engineering, Guiyang University, Guiyang 550005, China; 2 State Key Laboratory of Environmental Geochemistry, Institute of Geochemistry, Chinese Academy of Sciences (IGCAS), Guiyang 550081, China

**Keywords:** artificial soil, bioremediation, microbial diversity, recycling of solid waste, soil ecology

## Abstract

Red mud and phosphogypsum are two typical industrial by-products. The preparation of red mud/phosphogypsum-based artificial soils offers a promising novel solution to the efficient synergistic disposal of them. Fungi, as key drivers, can promote the continuous development and ecological improvement of artificial soils. This study is first to report the characteristics of fungal communities in three artificial soils after one year of incubation. The preliminary formation of fungal communities (with relatively low diversity) resulted in a total of 3 fungal phyla, 81 fungal genera, and 144 operational taxonomic units (OTUs) in artificial soils. Ascomycota was the dominant fungal phylum in each artificial soil (>99.5%), and the high-abundance fungal genera included *Unclassified_c_Sordariomycetes*, *Unclassified_o_Sordariales*, *Emericellopsis*, *Kernia*, *Unclassified_f_Nectriaceae*, *Ramophialophora*, *Schizothecium*, and *Iodophanus*. There were significant differences among the three artificial soils in the compositions of fungal genera, which affected material cycling, ecological succession, and soil development and maturation to varying extents. According to the FUNGuild prediction of fungal communities, saprotrophic fungi (such as undefined saprotroph, dung saprotroph–undefined saprotroph, and dung saprotroph) played dominant roles in promoting the degradation and humification of organic matter and the cycling of carbon in artificial soils. Fungal communities in the three artificial soils had strong correlations with many environmental factors (such as pH, organic matter, available nitrogen, total nitrogen, available phosphorous, sucrase, urease, acid phosphatase, alkaline phosphatase, and catalase), indicating significant interactions between them. This is not only conducive to the continuous optimization of the structure of fungal communities in artificial soils but also promotes the balanced and homogeneous distribution of various substances, promoting continuous soil development and maturation and gradual improvement in its ecological functions. This study provides an important scientific basis for clarifying the mechanisms of mycogenesis during the continuous development and maturation of artificial soils.

## Simple Summary

The preparation of artificial soil is a potential resource utilization scheme of red mud and phosphogypsum. Fungi can promote the continuous development and ecological improvement of the artificial soils. Here, we firstly report the characteristics of fungal communities in artificial soils; we initially found a total of 3 fungal phyla, 81 fungal genera, and 144 OTUs in artificial soils. Saprotrophic fungi played dominant roles in promoting the degradation and humification of organic matter and the cycling of carbon in artificial soils. Fungal communities in artificial soils had strong correlations with many environmental factors (such as pH, organic matter, available nitrogen, total nitrogen, available phosphorous, sucrase, urease, acid phosphatase, alkaline phosphatase, and catalase). This is not only conducive to the continuous optimization of the structure of fungal communities in artificial soils but also promotes the balanced and homogeneous distribution of various substances, promoting continuous soil development and maturation and the gradual improvement of its ecological functions.

## 1. Introduction

Red mud is a by-product of the alumina industry and is strongly alkaline (pH ~ 12). For every ton of alumina produced, approximately 1–2 tons of red mud are generated. The global cumulative yield of red mud has reached approximately 4 billion tons, with an annual increase of up to 120 million tons [1]. Phosphogypsum, on the other hand, is a strongly acidic (pH ~ 4) by-product of the phosphorus chemical industry. For every ton of phosphoric acid produced, approximately 4–5 tons of phosphogypsum are generated. The global cumulative yield of phosphogypsum has reached 7 billion tons, with an annual increase of up to 250 million tons [2]. Due to the huge amount of production and lack of effective disposal methods, red mud and phosphogypsum have been stockpiled for years, forming numerous yards that now pose serious problems [3,4].

Both red mud and phosphogypsum are highly corrosive and contain many pollutants such as heavy metals, sulfates, fluoride ions, and phosphates [5,6,7,8,9]. When there is a strong wind, red mud and phosphogypsum particles can easily form fugitive dust, causing corrosion as well as heavy metal poisoning when contacting the respiratory tracts and tissues of humans, animals, and plants [10,11]. When it rains, leachate discharged from red mud and phosphogypsum yards poses large toxicity risks to surrounding water bodies, soils, and environments, harming crop growth and the health of organisms [12,13]. As their cumulative yields continue to multiply with each passing day, stockpiled red mud and phosphogypsum are encroaching upon land resources on an increasingly large scale; the construction, maintenance, and management of red mud and phosphogypsum yards also pose tremendous challenges [14,15]. In cases of catastrophic weather such as geological disasters and continuous rainstorms, these yards may collapse, causing incalculable consequences. The long-term stockpiling and continuous addition of these substances have also posed huge risks and challenges to local social governance and government administration, as well as the sustainable development of the alumina and phosphorus chemical industries [16,17,18].

Red mud and phosphogypsum can engage in acid–alkali neutralization, and they both contain many beneficial elements (such as Si, Fe, Al, Ca, K, Mg, S, and P) commonly found in natural soil [5,19]. Red mud also shares the attributes of clay minerals [20]. Red mud/phosphogypsum-based artificial soils have been successfully prepared with red mud and phosphogypsum as raw materials and supplemented with other materials, and it has been preliminarily confirmed in our previous studies that they have great potential for the efficient synergistic recycling of red mud and phosphogypsum, especially in vegetation restoration and landscaping in mining areas [21,22]. However, it is worth noting that, as relatively primitive mixed substrates, artificial soils still need long-term evolution and continuous development before they become as mature, fertile, and stable as natural soils.

Microorganisms have stronger environmental adaptability and often colonize adverse habitats as pioneer organisms compared with animals and plants [23]. In the course of continuous succession, they manifest strong abilities to transform compounds, playing key roles in modifying the physicochemical properties of soils and the cycles of nutrients (such as N and P), improving the fertility of humic organic matter and the ecological functions of soil micro-environments [24,25,26]. In a previous study, we reported the diversity and functional characteristics of bacterial communities in three different treatments of red mud/phosphogypsum-based artificial soils; the dominant bacterial phyla such as Cyanobacteria, Proteobacteria, Actinobacteriota, and Chloroflexi were detected, as well as dominant bacterial genera such as *Microcoleus_PCC-7113*, *Rheinheimera*, and *Egicoccus*, which have very diverse functions such as photoautotrophy, chemoheterotrophy, aromatic compound degradation, fermentation, nitrate reduction, and cellulolysis [27]. These microbes are crucial for biological carbon/nitrogen fixation, organic decomposition, resistance to saline–alkali stress and pollutant toxicity, and enzyme activity enhancement in artificial soils.

As another soil microorganism, fungi are eukaryotes, differing from bacteria. They exist in a great variety and are widely found in soils, playing extensive roles in material cycling, energy flow, and soil ecology [26,28]. In particular, fungi exist as decomposers and symbionts, carry out multiple important ecological functions, and produce significant advantages in terms of improving soil physicochemical properties and fertility [29,30], decomposing plant residues and litter, promoting plant growth through symbiosis, and shaping rhizosphere soil micro-ecosystems [31]. Mycorrhizal fungi (such as arbuscular mycorrhizal fungi and ectomycorrhizal fungi) can drive soil carbon cycling by efficiently decomposing plant litter [32]. Endophytic fungi can enhance the environmental adaptability of ligneous plants, such as disease resistance, insect resistance, and heavy metal tolerance [33]. White-rot fungi secretions can alleviate soil salinization and promote seed germination and plant growth [30]. The composition of fungal communities in artificial soils, like that of bacterial communities, can also play a significant role in the natural succession, evolution, and continuous development of artificial soils, as well as in the adaptive growth of plants and the shaping of soil micro-ecosystems [34]. For example, in soil development, there is usually a process of gradual succession from bacteria to fungi, and even the biomass of fungi far exceeds that of bacteria, thus leading to improvements in soil environmental quality and ecological functions [35]. Building on our previous research [21,22], this study reported for the first time the compositional information of fungal communities in three different treatments of red mud/phosphogypsum-based artificial soils, providing an important scientific basis for clarifying the mechanisms of mycogenesis during the continuous development and maturation of artificial soils.

## 2. Materials and Methods

### 2.1. Preparation of Red Mud/Phosphogypsum-Based Artificial Soils

The red mud and phosphogypsum used in this study were collected from a red mud yard and a phosphogypsum yard in Guizhou Province, China, respectively; they had pH values of 11.4 and 1.8 and moisture contents of 23.1% and 21.9%, respectively. The auxiliary materials included common bentonite, fly ash, rice husk powder, and polyacrylamide flocculants. Based on the artificial soil preparation scheme with the above main and auxiliary materials in our previous research [21], three artificial soils (T7, T8, and T9) with higher organic matter content were selected from a total of nine artificial soils (T1~T9). The preparation process, which has been detailed in our previous paper [21,27], is briefly described as follows. Air-dried and sieved red mud and phosphogypsum were mixed in a mass ratio of 2.5:1 to form a neutral red mud/phosphogypsum substrate, which was then added to the above four auxiliary materials in different proportions (Table 1). After full mixing, 10 mL of a dilute suspension of microorganisms was added (mainly *Bacillus*, e.g., *Bacillus amyloliquefaciens*, *Bacillus licheniformis*, and *Bacillus subtilis*, with an effective viable bacterial count > 0.4 million/mL) to obtain the artificial soils, which were constantly watered to maintain a moisture content of about 30%.

### 2.2. Sample Collection and Determination

During the incubation and development process of artificial soil, a total of twelve plant species (including rapeseed, chili pepper, alfalfa, rye grass, wheat, clover, *Portulaca grandiflora*, lavender, *Gypsophila paniculata*, *Agrostemma githago*, globe amaranth, and red rose) were placed in the three artificial soils (T7~T9) three times, and seed germination and potted-seedling cultivation tests were conducted successively to verify the adaptability of different plants on the artificial soils (we confirmed that the three artificial soils had both high organic matter content and certain plant habitability, as detailed in our previous paper [21]). After 1 year, the artificial soil samples were collected, lyophilized, and crushed. They were then further sub-sampled to analyze basic physicochemical indicators (pH, moisture content (W_H2O_), organic matter (OM), cation-exchange capacity (CEC)), major nutrients (total nitrogen (TN), total phosphorous (TP), total potassium (TK), available nitrogen (AN), available phosphorous (AP), and available potassium (AK)), and enzyme activities (catalase (S-CAT), sucrase (S-SC), urease (S-UE), alkaline phosphatase (S-AKP), and acid phosphatase (S-AP)) [27], bacterial diversity [27], and fungal diversity. All samples were analyzed in three parallel sets.

The composition of fungal communities in the artificial soil samples was examined through high-throughput sequencing by Shanghai Majorbio Bio-pharm Technology Co., Ltd. (https://www.majorbio.com; accessed on 15 January 2025). DNA purity and the concentration of the samples were determined using a NanoDrop2000 (Thermo Fisher Scientific, Waltham, MA, USA). DNA integrity was tested through agarose gel electrophoresis (the samples were melted on ice, fully mixed, and centrifuged, and an appropriate amount was run on a 1% agarose gel at 5 V/cm for 20 min). For PCR, fungal primers (ITS1F_ITS2R) were designed as follows: ITS1F: CTTGGTCATTTAGAGGAAGTAA; ITS2R: GCTGCGTTCTTCATCGATGC. Initial denaturation was at 95 °C for 3 min, followed by 33 cycles of denaturing at 95 °C for 30 s, annealing at 55 °C for 30 s and extension at 72 °C for 45 s, and a single extension at 72 °C for 10 min.

### 2.3. Data Analysis

The presence of significant differences in the data was determined by a one-way analysis of variance and least significant difference analysis. The diversity of fungal communities in artificial soils and their correlations with different environmental factors (including Venn [36], Barplot [36], Alpha diversity [37], Beta diversity (PCoA) [38], Kruskal–Wallis H test [39], RDA/CCA [40], FUNGuild [41], Heatmap [42]) were analyzed and mapped (with the exception of Alpha diversity indices, which were mapped by Sigmaplot 10.0) on the online Majorbio Cloud Platform (www.majorbio.com; accessed on 15 January 2025).

## 3. Results

### 3.1. Composition Characteristics of Fungal Communities

**Figure 1:**
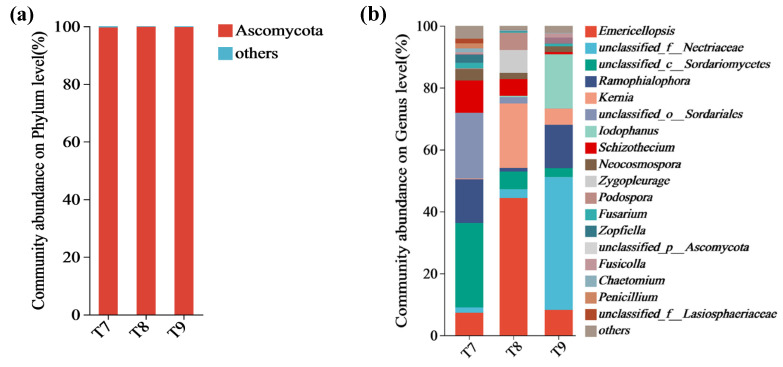
Composition of fungal communities (phyla (**a**), genera (**b**)) in artificial soils.

**Figure 2:**
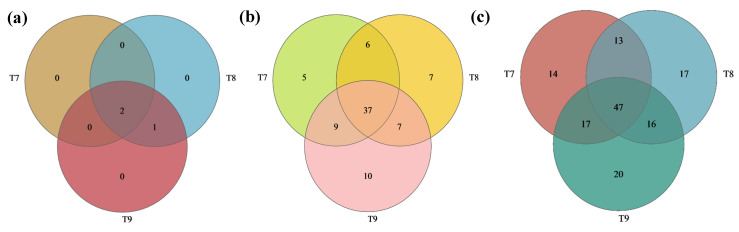
Venn analysis of fungal communities (phyla (**a**), genera (**b**), and OTUs (**c**)) in artificial soils.

The numbers of optimized sequences, valid sequences, and OTUs of artificial soil samples are shown in Table 2. A total of 3 fungal phyla, 81 fungal genera, and 144 operational taxonomic units (OTUs) were discovered in T7–T9. Ascomycota was the dominant fungal phylum in T7–T9, accounting for 99.6%, 99.8%, and 99.7%, respectively, while other fungal phyla (Basidiomycota and Mucoromycota) jointly accounted for below 0.5% each (Figure 1a). At the genus level, T7 was dominated by *Unclassified_c_Sordariomycetes*, *unclassified_o_Sordariales*, *Ramophialophora*, *Schizothecium*, and *Emericellopsis*, which accounted for 27.3%, 21.2%, 14.2%, 10.4%, and 7.3% of the total abundance, respectively (80.4% in total), while other fungal genera each accounted for less than 5% (Figure 1b). T8 was dominated by *Emericellopsis*, *Kernia*, *Zygopleurage*, *unclassified_c_Sordariomycetes*, *Podospora*, and *Schizothecium*, which accounted for 44.4%, 20.8%, 7.4%, 5.8%, 5.6%, and 5.4%, respectively (89.4% in total), while other fungal genera each accounted for less than 5% (Figure 1b). T9 was dominated by *Unclassified_f_Nectriaceae*, *Iodophanus*, *Ramophialophora*, *Emericellopsis*, and *Kernia*, which accounted for 43.0%, 17.5%, 14.0%, 8.2%, and 5.3%, respectively (88.0% in total), while other fungal genera each accounted for less than 5% (Figure 1b). The Venn analysis of fungal communities at the phylum level showed that there were two identical fungal phyla (66.7%) among T7, T8, and T9, and one identical fungal phylum between T8 and T9; none of the three soils had any unique fungal phyla (Figure 2a–c). At the genus level, there were 37 identical fungal genera among T7, T8, and T9 (accounting for 45.7%), and they had 5, 7, and 10 unique fungal genera, respectively. There were 47 identical OTUs among T7, T8, and T9 (accounting for 32.6%), and they had 14, 17, and 20 unique OTUs, respectively.

### 3.2. Alpha Diversity Indices of Fungal Communities

**Figure 3:**
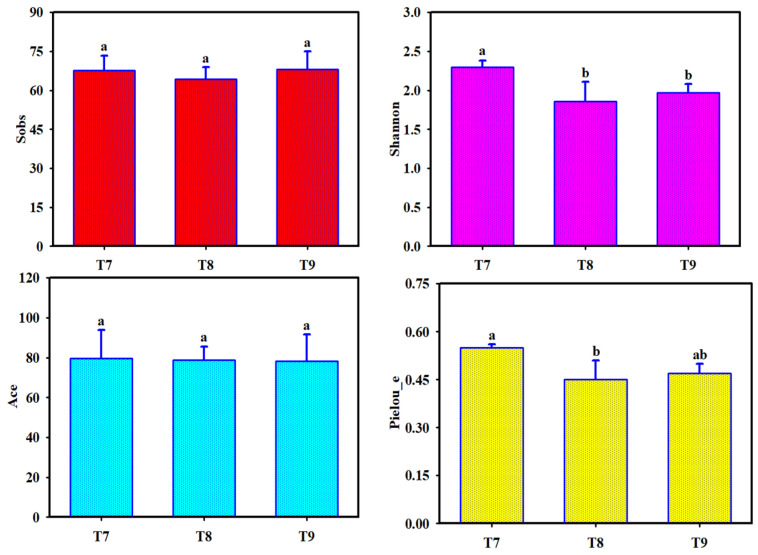
Alpha diversity indices of fungal communities in artificial soils. Note: Different letters represent significant differences among the three artificial soils.

Among the alpha diversity indices of fungal communities in T7–T9 (Figure 3), the Sobs and Ace indices ranged between 64.3 and 68.0 and 78.3 and 79.6, respectively, with no significant difference (*p* > 0.05), indicating that the abundances of fungi in the three artificial soils were very similar (Figure 1). The Shannon indices of T7–T9 ranged from 1.9 to 2.3; that of T7 was significantly higher than those of T8 and T9 (*p* < 0.05), whereas there was no significant difference between T8 and T9 (*p* > 0.05), that is, the fungal diversity was relatively higher in T7, compared to T8 and T9. The Pielou_e indices of T7–T9 ranged from 4.5 × 10^−1^ to 5.5 × 10^−1^; that of T7 was significantly higher than that of T8 (*p* < 0.05), whereas there was no significant difference between T7 and T9 or between T8 and T9 (*p* > 0.05). This indicated that the three artificial soils had similar evenness of fungal communities, but the overall evenness of fungal communities in T7 was slightly higher. The coverage indices of T7–T9 ranged from 0.9992 to 0.9994 (*p* > 0.05), indicating that almost all fungi in the three artificial soils could be detected and that the true compositions of the fungal communities were objectively reflected by the results.

### 3.3. Beta Diversity of Fungal Communities

**Figure 4:**
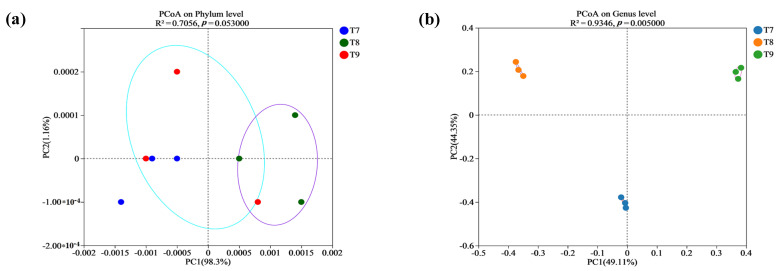
PCoA analysis of beta diversity of fungal communities (phyla (**a**), genera (**b**)) in artificial soils.

**Figure 5:**
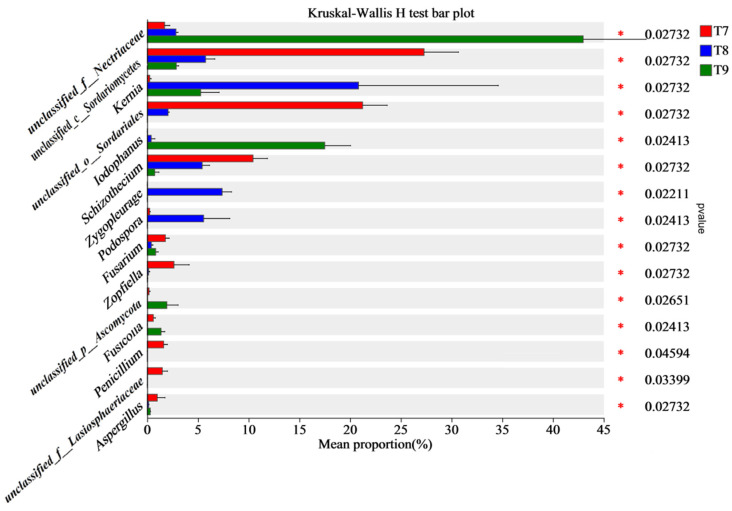
Kruskal–Wallis H test (*p*-values by taxonomic group) of fungal communities in artificial soils. Note: “*” means significant difference in the abundance of same fungus among the three artificial soils (*p* < 0.05).

At the phylum level, PCA1 and PCA2 upon principal coordinate analysis (PCoA) (R^2^ = 0.7056, *p* = 0.053) explained 98.3% and 1.16% of the total variation in the fungal community, respectively, totaling 99.46% (Figure 4a). The fungal phyla in T7–T9 were highly overlapped without independent partition, which confirmed the fact that Ascomycota were predominant among all the three artificial soils. However, at the genus level, PCA1 and PCA2 in PCoA (R^2^ = 0.9346, *p* = 0.005) explained 49.11% and 44.35% of the total variation in the fungal community, respectively, totaling 93.46% (Figure 4b). There were significant differences in fungal genera among the three artificial soils, and they all showed fine aggregations, which indicated the relatively stable structures of the fungal communities. At least 15 of the fungal genera showed significant differences among the three artificial soils (*p* < 0.05), including *Unclassified_f_Nectriaceae*, *unclassified_c_Sordariomycetes*, *Kernia*, *unclassified_o_Sordariales*, *Iodophanus*, *Schizothecium*, and *Zygopleurage* (Figure 5).

### 3.4. Functional Prediction of Fungal Communities

FUNGuild (Fungi Functional Guild) can classify fungal communities based on micro-ecological guilds. Fungal species belonging to the same category, regardless of the closeness of affinity, can utilize similar environmental resources in similar ways. Depending on their nutrition modes, fungi are generally classified into three categories: pathotrophic fungi, which obtain nutrients by damaging host cells (including phagocytic fungus phagotrophs); symbiotrophic fungi, which obtain nutrients by exchanging resources with host cells; and saprotrophic fungi, which obtain nutrients by degrading dead host cells. These three categories can be further divided into several guilds.

**Figure 6:**
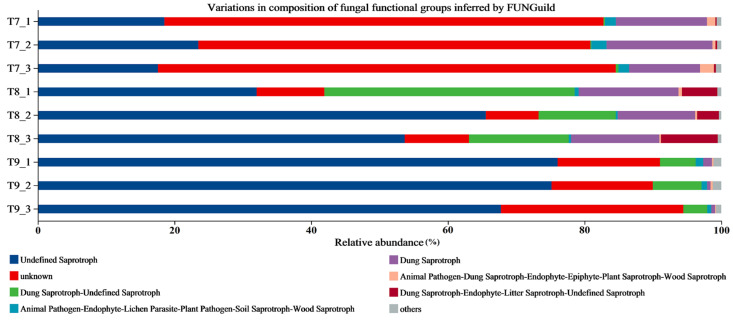
Composition of fungal functional groups inferred by FUNGuild in artificial soils.

In this study, there were more than eight fungal community guilds in T7–T9, including undefined saprotroph, unknown, dung saprotroph–undefined saprotroph, and dung saprotroph; however, there were some differences in the classification of fungal guilds between soil types (Figure 6). T7 was dominated by unknown, undefined saprotroph, and dung saprotroph, with relative abundances of 57.4–67.0% (average: 62.9%), 17.6–23.5% (19.9%), and 10.4–13.3% (13.1%), respectively, totaling 95.9%. T8 was dominated by undefined saprotroph, dung saprotroph–undefined saprotroph, unknown, and dung saprotroph, with relative abundances of 32.0–65.6% (50.4%), 11.3–36.6% (20.8%), 11.3–14.6% (13.0%), and 7.7–9.9% (9.0%), respectively, totaling 93.2%. T9 was dominated by undefined saprotroph, unknown, and dung saprotroph–undefined saprotroph, with relative abundances of 67.8–76.1% (73.0%), 14.8–26.7% (18.8%), and 3.5–7.1% (5.3%), respectively, totaling 97.1%.

### 3.5. Relationships Between Fungal Communities and Different Environmental Factors

**Figure 7:**
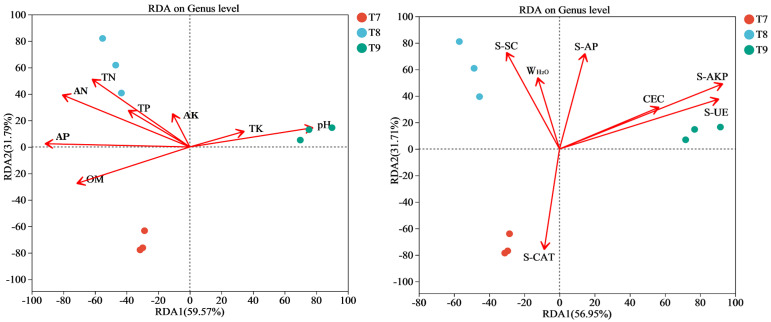
RDA/CCA analysis of fungal communities in artificial soils.

According to the RDA/CCA analysis of the relationships between fungi in T7–T9 and different environmental factors (Table 3 and Figure 7), fungal genera in T7 had different degrees of positive correlation with OM, AP, and S-CAT but were negatively correlated with W_H2O_, pH, TK, AK, S-SC, S-AP, CEC, S-AKP, and S-UE. Genera in T8 had different degrees of positive correlation with TN, AN, TP, AP, AK, OM, S-SC, W_H2O_, and S-AP but were negatively correlated with pH, TK, and S-CAT. Genera in T9 had different degrees of positive correlation with pH, TK, S-AP, CEC, S-AKP, and S-UE but were negatively correlated with AN, TN, TP, AP, OM, AK, and S-CAT.

**Figure 8:**
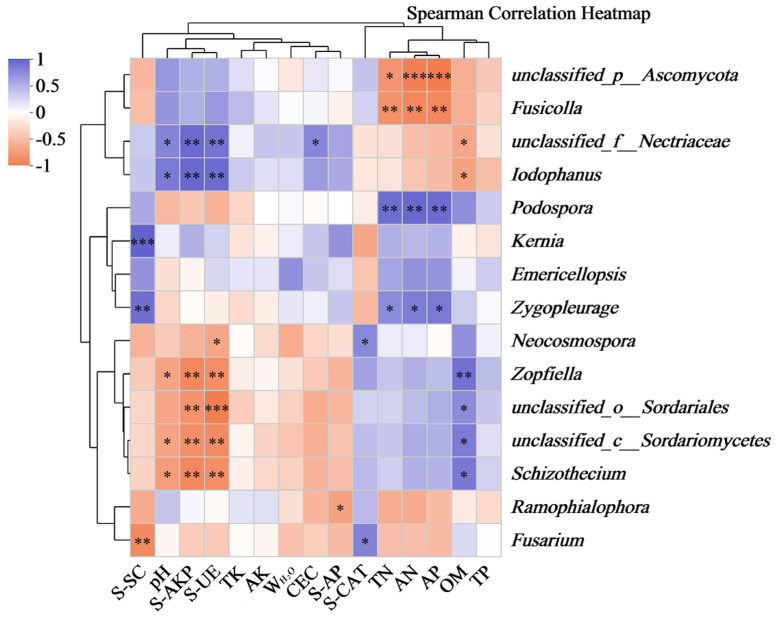
Spearman correlation heatmap analysis of fungal communities in artificial soils. (Note: * 0.01 < *p* ≤ 0.05; ** 0.001< *p* ≤ 0.01; *** *p* ≤ 0.001).

As shown in Figure 8, among the lower-level fungal taxa with an abundance of greater than 5%, *Unclassified_c_Sordariomycetes* and *Schizothecium* both showed significant negative correlations with pH, S-AKP, and S-UE but were positively correlated with OM; *Unclassified_o_Sordariales* showed a significant positive correlation with OM but significant negative correlations with S-AKP and S-UE; *Ramophialophora* showed a significant negative correlation with S-AP; *Kernia* showed a significant positive correlation with S-SC; *Zygopleurage* showed significant positive correlations with TN, AN, AP, and S-SC; *Podospora* showed significant positive correlations with TN, AN, and AP; *Unclassified_f_Nectriaceae* showed significant positive correlations with pH, S-AKP, S-UE, and CEC but a significant negative correlation with OM; and *Iodophanus* showed significant positive correlations with pH, S-AKP, and S-UE but a significant negative correlation with OM. Among the fungal genera with an abundance < 5%, *Unclassified_p_Ascomycota* and *Fusicolla* both showed significant or highly significant negative correlations with TN, AN, and AP; *Zopfiella* showed a significant positive correlation with OM but significant or highly significant negative correlations with pH, S-AKP, and S-UE.

## 4. Discussion

### 4.1. Fungi—Key Drivers of Artificial Soil Development and Ecological Enhancement

Preparing artificial soils with red mud and phosphogypsum as the main materials offers a novel technical solution with great application potential for vegetation restoration [21,22]. However, artificial soils prepared in this way are the relatively primitive mixed substrates of several materials and are still quite different from mature natural soils. The physicochemical properties and nutrient characteristics of these artificial soils fluctuate, and their key materials (such as rice husks) have not been completely humified; in addition, there are also risks such as high salinity and potential heavy metal pollution, potential poor adaptability of plants, low soil biodiversity, and unestablished micro-ecosystems [21,22]. Because of this, these relatively primitive artificial soils need to develop and mature to become truly similar to natural soils.

Fungi, like other microorganisms, are characterized by great variety and strong environmental adaptability [26,28,43]. Compared with higher animals and plants, they may show better adaptability and grow, reproduce, and metabolize naturally in soil more easily and can significantly enhance soil physical structure and bioactive substance [30,44]; they can accelerate the cycling, turnover, and redistribution of C, N, P, and enzyme activity; co-exist with plants and animals; gradually improve soil rhizosphere microenvironments; and construct soil ecosystems [45,46,47]. They constitute one of the key biological taxa that continuously drive the forward evolution, development, and maturation of artificial soils.

### 4.2. Formation and Functional Characteristics of Fungal Communities in Artificial Soils

The preliminary formation of fungal communities was observed in each artificial red mud and phosphogypsum soil; a total of 3 fungal phyla, 81 fungal genera, and 144 OTUs were identified. The identical fungal phyla, fungal genera, and OTUs between soils accounted for 66.7%, 45.7%, and 32.6%, respectively. Ascomycota was the dominant fungal phylum in each artificial soil (accounting for more than 99.5% in each case). As one of the most common fungal phyla, Ascomycota surpasses many eukaryotes of other taxa in terms of its ability to adapt to and occupy habitats [43,48,49]. When grown in a harsh soil environment, it can improve the soil and promote soil carbon cycling by degrading soil organic matter such as cellulose and lignin [50,51,52]. After colonizing the three artificial soils in the early phase, Ascomycota are expected to play important roles in improving artificial soil quality and boosting organic matter humification, carbon cycling, ecological function improvement, and pollutant degradation [53,54]. The other fungal phyla, such as Basidiomycota and Mucoromycota, each had an extremely low abundance (<0.5%), which to some extent also indicated that the three artificial soils were still in a primary stage of development, with a relatively simple fungal phyla and initial biodiversity [39].

The fungal genera in the three artificial soils were relatively abundant, differing greatly in species and abundance, which would help the fungal communities to deal with environmental stress from artificial soils and transform them through multiple pathways; they affected the material cycling, ecological succession, and developmental direction and speed of the three artificial soils to varying extents [55,56]. Each artificial soil had a certain abundance of *Emericellopsis*; it was the most abundant fungal genus in T8 (44.4%). *Emericellopsis* is generally considered a biologically functional fungal genus that can adapt well to high-salinity environments and produce bioactive substances; it is also an important source of bioactive compounds for soil [57]. Due to the high salinity of artificial soils and the relative scarcity of bioactive substances, *Emericellopsis* species are expected to play a key role in enriching bioactive substances and regulating soil biochemical reactions in artificial soils. In T7 and T8, there was a high abundance of *unclassified_c__Sordariomycetes*. *Sordariomycetes* is a large class of Ascomycota, characterized by high species diversity and wide distribution in various environmental media. It includes many endophytes, saprophytes, epiphytes, symbiotic fungi, and plant pathogens and can have extensive effects on organic matter decomposition, the symbiotic relationships between organisms, and diverse ecosystems in artificial soils [58,59,60]. In T9, the abundance of *Unclassified_f_Nectriaceae* reached 42.3%. There is also a great variety of *Nectriaceae*, including plant pathogens, endophytes, and saprophytes, which have strong environmental adaptability and play an important role in regulating soil ecology [61]. In T8, the abundance of *Kernia* reached 20.8%, which is expected to play a role in the biodegradation of pollutants in artificial soils [62].

From the perspective of the nutrition modes of fungi, saprotrophic fungi (such as undefined saprotroph, dung saprotroph–undefined saprotroph, and dung saprotroph) played dominant roles in the artificial soils, indicating that these fungi could decompose animal and plant residues or excrements to form inorganic nutrients for plants to utilize [63]. They may also play greater roles in the mobility of nutrients between organisms and the sustained stability of soil rhizosphere micro-ecosystems [63,64]. Although the three artificial soils were dominated by saprotrophic fungi, they had great differences in their abundance, which is likely to affect the decomposition, cycling, and turnover rates of compounds and further impact the development, fertility, and ecological succession characteristics of artificial soils [55,56]. It is worth mentioning that the proportion of fungal communities with unknown nutrition modes in T7 was as high as 62.9%, which is significantly higher than that in T8 and T9. Since this may have significant and unpredictable other effects on the development and ecology of artificial soil, further research into this difference is needed.

### 4.3. Relationship Between Fungal Communities and Different Environmental Factors in Artificial Soils

The compositions, abundance, and functional characteristics of fungal communities have very complicated relationships with different environmental factors in soils, including physical (such as temperature and humidity), chemical (such as pH [65], N and P [66], and organic matter content [67]), and biological factors (such as plant species, soil animals, and bacteria) [68,69]. Different environmental factors in soils regulate and optimize the compositional structure and abundance of fungal communities, which in turn diversify the biogeochemical cycling pathways of various substances in soils [70,71,72].

This study preliminarily indicated that fungal communities in the three artificial soils had correlations with many environmental factors (such as pH, OM, AN, TN, AP, S-SC, S-UE, S-AP, S-AKP, and S-CAT), which indicated the presence of significant interactions between them. This is not only conducive to the continuous optimization of the structure of fungal communities in artificial soils but also promotes the balanced and homogeneous distribution of various substances in artificial soils, the continuous improvement of soil fertility, and the improvement and stabilization of soil evolution. For example, Ascomycota, shared by the three artificial soils, showed a significant positive correlation with S-SC, indicating that it promoted the production of S-SC. This is because rice husks used to prepare artificial soils contain large quantities of cellulose and other sugars; S-SC, as a major sugar-decomposing enzyme, plays a very significant role in decomposing sugars in rice husks, improving the fertility and promoting the carbon cycling of artificial soils [50,52]. *Unclassified_c_Sordariomycetes*, *Unclassified_o_Sordariales*, and *Schizothecium*, each with a high abundance, were all positively correlated with OM. This suggests that organic matter in artificial soils can provide rich nutrients and saprophytic substrates for these fungal genera, which in turn promote the degradation and humification of organic matter and the cycling of carbon in artificial soils [73]. TN, AN, and AP were significantly correlated with some fungal genera, such as *Podospora*, *Zygopleurage*, *unclassified_p_Ascomycota*, and *Fusicolla*, indicating that N and P nutrients in artificial soils can regulate the abundance of these fungal genera, which in turn affect the conversion and cycling of N and P in artificial soils. Similarly, pH also regulated the structure of fungal communities in the artificial soils. Increased pH promoted the growth of *Unclassified_f_Nectriaceae* and *Iodophanus* but inhibited the activities of *Zopfiella*, *unclassified_c_Sordariomycetes*, and *Schizothecium*.

In addition, S-UE, S-AKP, S-AP, and S-CAT were also correlated with different fungal genera, indicating the direct or indirect effects of these fungal genera on enzyme activity in artificial soils [74,75]. *Unclassified_f_Nectriaceae* and *Iodophanus* enhanced the activity of S-UE and S-AKP, which facilitated the decomposition and cycling of nitrogenous and phosphorous substances in artificial soils [76,77]; *Neocosmospora* and *Fusarium* enhanced the activity of S-CAT, which improved the ability of artificial soils to catalyze the decomposition of hydrogen peroxide [78]; some fungi (*Zopfiella*, *unclassified_c_Sordariomycetes*, *Unclassified_o_Sordariales*, *Schizothecium*) and the two enzymes (S-UE and S-AKP) showed mutual inhibition, which might have involved the complex metabolic behaviors of some fungi, the competition with other microorganisms for nutrients, and overall regulation by the external environment [79,80,81].

## 5. Conclusions

The preparation of artificial soils offers a promising novel solution to the efficient synergistic disposal of red mud and phosphogypsum. Fungi can be key drivers for the continuous development and ecological enhancement of artificial soil. This study reported for the first time the characteristics of fungal communities in three artificial soils after one year of incubation. The preliminary formation of fungal communities (with relatively low diversity) resulted in a total of 3 fungal phyla, 81 fungal genera, and 144 OTUs. Ascomycota was the dominant fungal phylum in each artificial soil (>99.5%), and the high-abundance fungal genera included *Unclassified_c_Sordariomycetes*, *Unclassified_o_Sordariales*, *Emericellopsis*, *Kernia*, *Unclassified_f_Nectriaceae*, *Ramophialophora*, *Schizothecium*, and *Iodophanus*. There were significant differences among the three artificial soils in the compositions of fungal genera, which affected material cycling, ecological succession, and soil development and maturation to varying extents. According to the FUNGuild prediction of fungal communities, saprotrophic fungi (such as undefined saprotroph, dung saprotroph–undefined saprotroph, and dung saprotroph) played dominant roles in promoting the degradation and humification of organic matter and the cycling of carbon in artificial soils. Fungal communities in the three artificial soils had strong correlations with many environmental factors (such as pH, OM, AN, TN, AP, S-SC, S-UE, S-AP, S-AKP, and S-CAT), indicating significant interactions between them. This is not only conducive to the continuous optimization of the structure of fungal communities in artificial soils but also promotes the balanced and homogeneous distribution of various substances, promoting continuous development and maturation of the soil and the gradual improvement of its ecological functions. In the future, long-term and frequent synchronous monitoring and coupling analyses of fungal communities and different environmental factors such as physical and chemical indexes, nutrient elements, enzyme activities, and heavy metals in artificial soils are needed to explore the co-evolutionary relationship between the fungal community and artificial soil’s environmental quality. Moreover, it is necessary to analyze bacterial diversity, arthropod communities, and above-ground vegetation in artificial soil to explore the succession behavior of the fungal community, the symbiosis of different organisms, and the construction mechanism of the rhizosphere micro-ecosystem in artificial soil.

## Figures and Tables

**Table 1:** Compositions of red mud/phosphogypsum-based artificial soils [27].

Name	Red Mud–Phosphogypsum Substrate (g)	Rice Husk Powder (g)	Bentonite (g)	Fly Ash (g)	Polyacrylamide Flocculant (g)
T7	200	20	4	10	0.5
T8	200	20	10	2	1
T9	200	20	20	5	0.25

**Table 2:** Numbers of optimized sequences, valid sequences and OTUs of artificial soil samples.

Name	Seq-Optimized	Seq-Valid	OTUs
T7_1	38,517	20,998	66
T7_2	33,173	20,998	74
T7_3	47,092	20,998	63
T8_1	33,306	20,998	67
T8_2	66,568	20,998	67
T8_3	58,221	20,998	59
T9_1	32,293	20,998	60
T9_2	37,599	20,998	72
T9_3	31,873	20,998	72

Note: Seq-optimized and Seq-valid refer to optimized sequences and valid sequences, respectively.

**Table 3:** Basic physicochemical indicators, major nutrients, and enzyme activities of artificial soils [27].

Name	pH	W_H2O_ (%)	OM (%)	CEC (cmol/kg)	TN (mg/kg)	TP (mg/kg)	TK (g/kg)	AN (mg/kg)
T7	8.4 ± 0.1 b	30.1 ± 1.5 a	5.5 ± 0.1 a	11.1 ± 0.3 a	445.5 ± 52.2 b	2726.4 ± 196.8 a	31.8 ± 1.8 a	153.6 ± 22.0 b
T8	8.4 ± 0.0 b	31.7 ± 2.1 a	5.4 ± 0.3 a	11.4 ± 0.4 a	596.0 ± 83.8 a	2842.9 ± 454.7 a	32.4 ± 4.4 a	211.4 ± 8.2 a
T9	8.7 ± 0.1 a	30.9 ± 1.8 a	5.0 ± 0.1 b	12.3 ± 1.5 a	395.2 ± 80.4 b	2578.6 ± 108.3 a	38.6 ± 11.9 a	110.5 ± 8.3 c
**Name**	**AP** **(mg/kg)**	**AK** **(mg/kg)**	**S-CAT** **(ml/g)**	**S-AKP** **(mg/g, 24 h)**	**S-UE** **(mg/g, 24 h)**	**S-AP** **(mg/g, 24 h)**	**S-SC** **(mg/g, 24 h)**	
T7	482.7 ± 42.4 b	14.2 ± 0.6 a	1.285 ± 0.071 a	0.329 ± 0.049 c	0.318 ± 0.058 b	0.028 ± 0.002 a	1.020 ± 0.238 b	
T8	548.2 ± 19.5 a	15.4 ± 4.3 a	1.121 ± 0.121 a	0.417 ± 0.039 b	0.425 ± 0.072 b	0.030 ± 0.002 a	1.762 ± 0.385 a	
T9	180.8 ± 31.7 c	14.5 ± 0.9 a	1.162 ± 0.073 a	0.561 ± 0.004 a	0.789 ± 0.176 a	0.029 ± 0.004 a	1.253 ± 0.008 b	

Note: Different letters represent significant differences among the three artificial soils.

## Data Availability

The original contributions presented in the study are included in the article; further inquiries can be directed to the corresponding author.
